# Scalable Gromov–Wasserstein Based Comparison of Biological Time Series

**DOI:** 10.1007/s11538-023-01175-y

**Published:** 2023-07-07

**Authors:** Natalia Kravtsova, Reginald L. McGee II, Adriana T. Dawes

**Affiliations:** 1 Department of Mathematics, The Ohio State University, 231 West 18th Avenue, Columbus, OH 43210 USA; 2 Department of Mathematics and Computer Science, College of the Holy Cross, 1 College Street, Worcester, MA 01609 USA; 3 Department of Molecular Genetics, The Ohio State University, 484 West 12th Avenue, Columbus, OH 43210 USA

**Keywords:** Gromov–Wasserstein distance, Time series distance, Optimal transport, Biological time series

## Abstract

A time series is an extremely abundant data type arising in many areas of scientific research, including the biological sciences. Any method that compares time series data relies on a pairwise distance between trajectories, and the choice of distance measure determines the accuracy and speed of the time series comparison. This paper introduces an optimal transport type distance for comparing time series trajectories that are allowed to lie in spaces of different dimensions and/or with differing numbers of points possibly unequally spaced along each trajectory. The construction is based on a modified Gromov–Wasserstein distance optimization program, reducing the problem to a Wasserstein distance on the real line. The resulting program has a closed-form solution and can be computed quickly due to the scalability of the one-dimensional Wasserstein distance. We discuss theoretical properties of this distance measure, and empirically demonstrate the performance of the proposed distance on several datasets with a range of characteristics commonly found in biologically relevant data. We also use our proposed distance to demonstrate that averaging oscillatory time series trajectories using the recently proposed Fused Gromov–Wasserstein barycenter retains more characteristics in the averaged trajectory when compared to traditional averaging, which demonstrates the applicability of Fused Gromov–Wasserstein barycenters for biological time series. Fast and user friendly software for computing the proposed distance and related applications is provided. The proposed distance allows fast and meaningful comparison of biological time series and can be efficiently used in a wide range of applications.

## Introduction

Time series, where observations are organized into a discrete, ordered list or trajectory, is one of the most important data types across many biological disciplines, including genetics (Bar-Joseph et al. 2012), epidemiology (Bhaskaran et al. 2013), ecology (Turchin and Taylor 1992), and medical sciences (Wei et al. 2005). Comparing biological time series within and between different groups of subjects or different experimental conditions allows for the identification of features associated with a group/condition of interest. Any comparison of time series relies on a pairwise dissimilarity measure between trajectories, with a large body of literature devoted to various types of pairwise distances^1^ (see Wang et al. 2013 for a review of distance measures). A distance measure is required for any type of machine learning task that quantitatively compares time series (Esling and Agon 2012), such as time series classification (Abanda et al. 2019) or clustering (Aghabozorgi et al. 2015). Thus, the type of distance chosen for a particular machine learning task determines the accuracy and speed of time series comparison (Ding et al. 2008).

Here, we propose a time series distance measure that captures differences in overall shapes of the trajectories, making the measure applicable to a wide range of biological time series datasets. This new distance measure is especially suited to time series trajectories whose shapes are indicative of underlying mechanisms or behavior. The idea behind this construction is to view trajectories in the dataset as separate metric spaces, and compare these metric spaces with the aid of optimal transport. The general principle of viewing each member of (any) dataset as its own metric space and then utilizing optimal transport to compare the metric spaces has been an active area of research in the past decade, with significant progress achieved in both the theoretical principles and application of these concepts. The pioneering work of Mémoli (2011) defined a distance between metric spaces termed the *Gromov-Wasserstein (GW)* distance, which can be used to distinguish between objects with different shapes. In practical terms, the construction requires each object in the dataset to be viewed as a separate metric space under (some) intrinsic distance (*gromovization*) with some defined measure; the comparison is made by finding an optimal probabilistic correspondence between intrinsic distances within each space using these defined measures.

The gromovization principle proves extremely useful when comparing objects that are not easily embeddable in a common space, which is frequently the case for biological data due to, for instance, missing data points or observations recorded on different time scales. GW comparison frameworks and gromovization have recently been applied to specific biologically-based problems, including analysis of protein-protein networks (Xu et al. 2019a, b), alignment of single-cell multi-omics datasets (Demetci et al. 2022), and determination of protein structures (Weitkamp et al. 2022), building on recent developments in GW-type constructions (Peyré et al. 2016; Chowdhury and Mémoli 2019).

For general time series data, gromovization was recently proposed in Cohen et al. (2021), where a time series distance termed *Gromov dynamic time warping (GDTW)* is defined as the minimal cost of matching intrinsic distances of two trajectories for all pairs of points inside each trajectory. The matching is performed in a prescribed manner by finding an optimal alignment matrix whose entries only have values in $\{1,0\}$ (match/no match, respectively). The problem is efficiently solved by a proposed Frank-Wolfe-type iterative algorithm, and the resulting distance is used in further applications, in particular for the classical problem of barycenter averaging (Peyré et al. 2016). While our approach is also based on gromovization, our matching principle is different from Cohen et al. (2021): instead of a binary $\{1,0\}$ correspondence, we use a probabilistic correspondence between measures that we define on the trajectories, as originally proposed for GW distance construction in Mémoli (2011). Furthermore, instead of comparing all intrinsic distances of two trajectories as would be done when computing GW or GDTW, we fix one coordinate in the intrinsic distance function for each trajectory and only compare intrinsic distances from the start of each trajectory. This places our construction in a *tree Gromov-Wasserstein* context, an area of current active research in mathematics and machine learning (see Section 6 of Le et al. 2021 for further discussion).

We term our construction $GW_\tau$ (with *GW* referring to the Gromov-Wasserstein framework and $\tau$ referring to both *time series* and *tree*). Assigning each trajectory the vector of distances from the start of a time series relates $GW_\tau$ to *distance histogram functions* defined for planar curves in Brinkman and Olver (2012) and *local distribution of distances* defined for any metric-measure spaces in Mémoli (2011). The probabilistic comparison of vectors of intrinsic distances from the start of each trajectory relates our $GW_\tau$ distance to the construction proposed in Le et al. (2021) that compares measures supported on (general) tree metric spaces, termed *aligned-root FlowAlign*.

The construction of *aligned-root FlowAlign* is defined in Le et al. (2021) for the case of discrete measures (and particular choice of the exponent $p=2$) as a special case (and a practical subroutine) of a more general construction *FlowAlign*. Aligned-root FlowAlign utilizes the tree structure of measure supports, allowing for efficient comparison. Constructions in Le et al. (2021) are shown to outperform the alternative methods in terms of speed and accuracy when applied for comparing tree-supported measures in problems including prediction of atomization energies of molecules in quantum chemistry, and classification of documents in machine learning. Our construction $GW_\tau$ has several distinct properties: it is (1) defined for general measures rather than discrete ones (and for a general exponent $p \in [1, \infty )$), (2) specific to time series, and (3) computed in linear time in the case when two time series have the same number of points (and quadratic time when they have a different number of points). The benefit of property (1) is the potential for statistical inference (such as testing for similarity between two trajectories) on the true value $GW_\tau$ between two trajectories, based on the empirical version computed from the data. We leave the application of $GW_\tau$ for statistical inference for future work. Similar to *aligned-root FlowAlign*, the empirical version of $GW_\tau$ has a closed-form solution; we note that in the specific case of time series (property (2)), it can be computed even faster than the general case complexity reported in Le et al. (2021) (property (3)), providing a scalable way to compare (possibly long) biological time series.

**Fig. 1:**
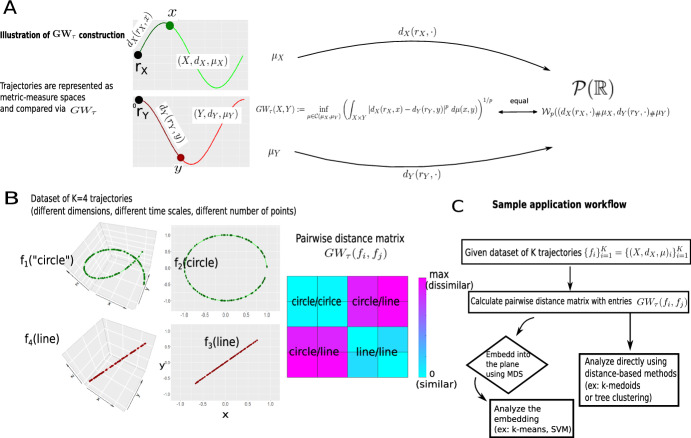
Illustration of $GW_\tau$ construction and sample applications. **A** viewing trajectories (green, top panel and red, bottom panel) as metric-measure spaces, we construct the distance $GW_\tau (X,Y)$ between them. Pushing the measures $\mu _X$ and $\mu _Y$ forward to $\mathbb {R}$ by the functions $d_X(r_X,\cdot )$ and $d_Y(r_Y,\cdot )$ results in the equivalence of $GW_\tau$ with the easily computable Wasserstein distances between the pushforwards (Proposition 1a). **B** illustration of Example 1: sample dataset of four trajectories lying in different dimensions, defined on different time scales, and having a different number of unequally spaced discrete time points. A distance matrix graphically summarizes $GW_\tau$ distances between pairs of trajectories. Trajectories with similar shapes are found to be similar (cyan), even though they lie in different dimensions. **C** proposed application workflow for $GW_\tau$, applied in Sect. 3 (Color figure online)

The paper is organized as follows. The Sect. 2 defines $GW_\tau$ (Definition 1) and its empirical version for the discrete data case (Definition 2). Theoretical properties of $GW_\tau$ and its relation to other constructions in the literature, as well as its computational complexity in the empirical case, are discussed in Proposition 1 and illustrated in Example 1 and Fig. 1. A sample application workflow using our $GW_\tau$ distance to compare biological trajectories is shown in Fig. 1C. The Sect. 3 provides an empirical evaluation of the performance of $GW_\tau$ on time series from three distinct sources: synthetic data, biological model based data, and publicly available datasets of physical measurements (Sect. 3.1). All of these time series datasets exhibit diverse features commonly found in biologically-based data. Further, $GW_\tau$ is applied to recently collected quantitative microscopy data (Ignacio et al. 2022) (Sect. 3.2). We demonstrate that $GW_\tau$ reliably distinguishes groups of trajectories belonging to different experimental conditions, in contrast to other commonly used time series distance measures. Finally, we apply $GW_\tau$ to show that averaging trajectories of this dataset via Fused Gromov-Wasserstein (FGW) barycenters (Vayer et al. 2020) produces barycenter curves that preserve the shape of the trajectories more accurately than the traditional method of calculating mean trajectories. This demonstrates the applicability of the FGW barycenter procedure in the context of biological time series, as first suggested in Vayer et al. (2020) for general time series case. The results of the paper demonstrate applicability of $GW_\tau$ for a wide range of time series analysis tasks and allow fast and meaningful comparison of biological time series data.

## Methods

### Notation

We define a *trajectory* as the image of an injective map^2^$f: [a,b] \rightarrow \mathbb {R}^d$, $f: t \rightarrow \bigl (f_1(t), \ldots , f_d(t)\bigr )$ whose coordinate functions $f_1, \ldots , f_d$ are continuously differentiable.^3^ In this work we consider trajectories in the plane ($d=2$) or in space $(d=3)$. We leave modeling biological processes with higher dimensional trajectories for future work. Viewing a trajectory as a path in the plane or in space, we adopt the following convention: the trajectory given by $y=h(t)$ is represented by the map $f: t \rightarrow \bigl (t, h(t)\bigr )$ with image in $\mathbb {R}^2$, and the trajectories given by $t \rightarrow \bigl (f_1(t),f_2(t)\bigr )$ and $t \rightarrow \bigl (f_1(t),f_2(t), f_3(t)\bigr )$ lie in $\mathbb {R}^2$ and $\mathbb {R}^3$, respectively.^4^ The length of a trajectory is given by $\int _a^b \Vert \dot{f}(t) \Vert \, dt$ and corresponds to $\int _a^b \sqrt{1 + h'(t)^2} \, dt$, $\int _a^b \sqrt{f_1'(t)^2 + f_2'(t)^2} \, dt$, and $\int _a^b \sqrt{f_1'(t)^2 + f_2'(t)^2 + f_3'(t)^2} \, dt$ in three cases, respectively.

By *time series* we mean a finite ordered list of points $\{\bigl (t_i,h(t_i)\bigr )\}_{i=1}^n$ (“1D time series”), $\{\bigl (f_1(t_i),f_2(t_i) \bigr ) \}_{i=1}^n$ (“2D time series”), and $\{\bigl (f_1(t_i),f_2(t_i),f_3(t_i) \bigr ) \}_{i=1}^n$ (“3D time series”). Time series can be interpreted as a finite collection of points from an image of an underlying injective map $f: t \rightarrow \bigl (f_1(t), \ldots , f_d(t) \bigr )$, $d \in \{ 2, 3 \}$.^5^ The length of the time series is given by the sum of lengths of line segments joining the points, i.e. $\sum _{i=1}^{n-1} \Vert f(t_{i+1}) - f(t_i)) \Vert$.

Throughout, we use the term *trajectory* for the discrete case of time series as well to highlight the relation between an observed time series data and possible underlying map *f*. We make it clear from the context whether trajectory is assumed discrete or continuous. We provide more detailed background information and discuss principles underlying construction of $GW_\tau$ for the continuous case (Sect. 2.2), followed by definition of $GW_\tau$ and discussion of properties for both continuous and discrete cases (Sect. 2.3).

### Background Definitions

A trajectory induced by $f: [a,b] \rightarrow \mathbb {R}^d$ is a metric space with points $X:= \{x \in \mathbb {R}^d: x=(f_1(t), \ldots , f_d(t)), t \in [a,b]\}$ under the intrinsic distance between any two points $x'=f(t_1)$ and $x=f(t_2)$, $t_1 \le t_2$, given by the length of the arc joining the two points, i. e.

1 $$\begin{aligned} d_X(x',x) = \int _{t_1}^{t_2} \Vert \dot{f}(t) \Vert \, dt \end{aligned}$$

and $d(x,x') = d(x',x)$. Similarly, for $g: [a,b] \rightarrow \mathbb {R}^k$,

2 $$\begin{aligned} d_Y(y',y) = \int _{t_1}^{t_2} \Vert \dot{g}(t) \Vert \, dt \end{aligned}$$

resulting in the metric spaces $(X, d_X)$ and $(Y, d_Y)$, respectively (Fig. 1A). Note that the distance function $d_X(\cdot ,\cdot )$ is well defined due to injectivity assumption on *f* made in Sect. 2.1. In order to compare these metric spaces under the Gromov-Wasserstein framework, one needs to turn these metric spaces into metric-*measure* spaces by defining Borel probability measures $\mu _X$ on *X* and $\mu _Y$ on *Y*. In practical terms, these measures essentially serve as “helpers” to make a comparison between $(X, d_X)$ and $(Y, d_Y)$ more computationally tractable, and conceptually they may be interpreted as markers of importance of certain regions of an underlying space (see the discussion on p. 440 of Mémoli (2011)). Here we assume that $\mu _X$ and $\mu _Y$ are defined on *X* and *Y* as fully supported Borel probability measures.

Equipped with distances and measures for each trajectory *f* and *g*, we represent the trajectories as metric-measure spaces $f = (X,d_X,\mu _X)$ and $g = (Y,d_Y,\mu _Y)$ (which we denote *X* and *Y*). These spaces can be compared using the *p*-Gromov-Wasserstein distance (Mémoli 2011):

3 $$\begin{aligned} GW_p(X, Y){} & {} := \frac{1}{2} \inf _{\mu \in \mathcal {C}(\mu _X, \mu _Y)} \nonumber \\{} & {} \quad \left( \int _{X\times Y} \int _{X\times Y} \arrowvert d_X(x',x) - d_Y(y',y)\arrowvert ^p \, d\mu (x',y') d\mu (x,y)\right) ^{1/p} \end{aligned}$$

where the constraint set $\mathcal {C}(\mu _X,\mu _Y)$ is the set of all *couplings* between $\mu _X$ and $\mu _Y$, i.e. the set of all Borel probability measures on a product space $X \times Y$ whose marginals are $\mu _X$ and $\mu _Y$. $GW_p$ defines a true distance between equivalence classes (up to measure-preserving isomorphism) of compact metric-measure spaces (see Theorem 5.1 of Mémoli (2011)) and thus can be used in applications to distinguish objects by representing them as metric-measure spaces and comparing via Gromov-Wasserstein distance. The expression (3) can be interpreted as the discrepancy between intrinsic distances $d_X(\cdot ,\cdot )$ on *X* and $d_Y(\cdot ,\cdot )$ on *Y* after the spaces *X* and *Y* are aligned in the best possible way. Larger values of this “best case" discrepancy indicate that it is more difficult to align the spaces (and hence the spaces are more different), and smaller values indicate that the spaces are more easily aligned (and hence more similar). Zero discrepancy indicates that the spaces are isomorphic, i.e. their points are in a 1-to-1 and onto correspondence, and such a correspondence preserves the measures.

Equation (3) results in a non-convex quadratic optimization program. For the case of a discrete time series, the program in (3) becomes

4 $$\begin{aligned} GW_p(X, Y) = \frac{1}{2} \inf _{\mu \in \mathcal {C}(\mu _X, \mu _Y)} \left( \sum _{(x,y) \in X\times Y} \sum _{(x',y') \in X\times Y} \arrowvert d_X(x',x) - d_Y(y',y) \arrowvert ^p \, \mu (x',y') \mu (x,y) \right) ^{1/p} \nonumber \\ \end{aligned}$$

which can only be solved using local methods with no guarantee of finding the true global minimum as required by Eq. (4) (Peyré et al. 2019). To overcome this problem, two main directions are currently taken in the literature: (1) add a regularization term to the right hand side of Eq. 4 which turns the problem into a sequence of convex programs (see Peyré et al. 2016 for details), or, (2) replace *GW* from the general definition in Eq. (3) by an easier-to-compute entity and demonstrate it works well with practical data applications. This second approach was taken in Mémoli (2011) and Chowdhury and Mémoli (2019) for comparison of metric-measure spaces and measure networks, respectively, where several constructions bounding *GW* (Eq. 3) from below were defined, termed *Lower Bounds (LB)’s* of GW. These lower bounds result in linear programs (or sequences thereof) that can be solved exactly and in (at most) polynomial time.

Here we adopt the second approach and propose to replace *GW* defined in Eq. (3) by the construction given in Definition 1 that we term $GW_\tau$. We provide an empirical version of $GW_\tau$ in the case of real-world data (Definition 2), discuss its properties (Proposition 1) and show a sample computation (Example 1). Figure 1 illustrates our construction and its properties.

### Definition of the Distance $GW_\tau$ Between Two Trajectories

#### Definition 1

($\mathbf {GW_\tau },$ **general case**) Let $f=(X,d_X,\mu _X)$ and $g=(Y,d_Y,\mu _Y)$ be two trajectories we wish to compare. Consider the distance function of two arguments $d_X(x',x)$ defined in Eq. (1), and fix the first (WLOG) argument $x'$ at the initial point of the trajectory $r_X:= f(a)$, producing the function $d_X(r_X,x)$ of a single argument *x*, representing the distance of a given point *x* from the start of the trajectory *f*. Similarly, $r_Y$ for $d_Y(r_Y,y)$ of the trajectory *g*. Let

5 $$\begin{aligned} GW_\tau (X,Y):= \inf _{\mu \in \mathcal {C}(\mu _X,\mu _Y)} \left( \int _{X \times Y} \arrowvert d_X(r_X,x) - d_Y(r_Y,y) \arrowvert ^p \, d\mu (x,y) \right) ^{1/p} \end{aligned}$$

*Notation* For notation simplicity, we drop the dependence on *p* from the name of $GW_\tau$. The dependence on *p* is implied, and we comment on particular values of *p* and its relevance to individual applications.

*Interpretation* Expression (5) can be interpreted as measuring discrepancy between intrinsic distances $d_X(r_X,\cdot )$ on *X* and $d_Y(r_Y,\cdot )$ on *Y* from the start of each trajectory. A larger discrepancy indicates that intrinsic distances differ more, and hence the trajectories are thought to be more different for larger values of $GW_\tau$.

*Note* the absence of the factor of $\frac{1}{2}$ in the definition of $GW_\tau$ compared to the definition of *GW*. This is merely for ease of interpretation, and this benefit is exploited in Proposition 1(b).

#### Definition 2

($\mathbf {GW_\tau },$ **discrete case (empirical version)**) In the discrete case, the trajectories are finite metric spaces $f = (X,d_X,\mu _X)$ and $g = (Y,d_Y,\mu _Y)$ with points

6 $$\begin{aligned} X:=\{i \in \mathbb {R}^d: x_i=f(t_i), i=1,\ldots , n \} \end{aligned}$$

and

7 $$\begin{aligned} Y:=\{y_j \in \mathbb {R}^k: y_j=g(t_j), j=1,\ldots , m \} \end{aligned}$$

The distance between any two points $x'=f(t_i)$ and $x=f(t_j)$ (WLOG, $j \ge i$) is given by

8 $$\begin{aligned} d_X(x', x)=\sum _{k=i}^{j-1} \left\| f(t_{k+1})-f(t_k) \right\| \end{aligned}$$

and

9 $$\begin{aligned} d_Y(y', y)=\sum _{k=i}^{j-1} \left\| g(t_{k+1})-g(t_k) \right\| \end{aligned}$$

respectively^6^, where $\Vert \cdot \Vert$ denotes the Euclidean distance on $\mathbb {R}^d$ (i.e. the distance between any two points is the length of the polygonal arc between them).

Define the measures on *X* and *Y* by

$$\begin{aligned} \mu _X=\sum _{i=1}^n \frac{1}{n} \delta _{x_i} \text { and } \mu _Y=\sum _{j=1}^m \frac{1}{m} \delta _{y_j} \end{aligned}$$

where $\delta _{x_i}$ denotes the delta function that evaluates to 1 if the data point equals $x_i$ and to 0 otherwise (i.e., we have discrete uniform probability measures).

Let

10 $$\begin{aligned} GW_\tau (X, Y) = \inf _{\mu \in \mathcal {C}(\mu _X, \mu _Y)} \left( \sum _{(x,y) \in X\times Y} \arrowvert d_X(r_X,x) - d_Y(r_Y,y)\arrowvert ^p \, \mu (x,y) \right) ^{1/p} \end{aligned}$$

*Note* The measures can be defined as general (rather than uniform) discrete probability measures $\mu _X=\sum _{i=1}^n a_i \delta _{x_i}$ and $\mu _Y=\sum _{j=1}^m b_j \delta _{y_j}$ by weighting the points along the time series differently according to some criteria suitable for the comparison of interest (for example, one can place higher weights for more important regions). We leave the investigation how different choices of measures affect time series comparison for future work.

#### Proposition 1

Consider trajectories of the form $f=(X,d_X,\mu _X)$ and $g=(Y,d_Y,\mu _Y)$, and let $GW_\tau$ be as in Definition 1 whose empirical version is given by Definition 2. Parts (a) - (c) concern both $GW_\tau$ and its empirical version; parts (d) and (e) concern the empirical version only.

- **Metric properties:** $GW_\tau (\cdot ,\cdot )$ is a distance on $\mathcal {S}:= \{\nu _X \in \mathcal {P}(\mathbb {R}): \, \nu = d_X(r_X,\cdot )_\# \mu _X \}$, the space of pushforwards of ground measures $\mu _X$. More precisely, $GW_\tau (X,Y)$ is a Wasserstein distance between measure pushforwards $d_X(r_X,\cdot )_\# \mu _X$ and $d_Y(r_Y,\cdot )_\# \mu _Y$.
- **Relation to Gromov-Wasserstein distance:** For any pair of trajectories $(X,d_X,\mu _X)$ and $(Y,d_Y,\mu _Y)$, we have that $GW(X,Y) \le GW_\tau (X,Y)$ for any $p \in [1, \infty )$.
- **Relation to local distributions of distances from **Mémoli (2011): $GW_\tau$ compares local distributions of distances at the start of each trajectory via Wasserstein distance.
- **Relation to** ***aligned-root FlowAlign*** ***from ***Le et al. (2021): The empirical version of $GW_\tau$ (Definition 2) is equivalent to the *aligned-root FlowAlign* construction (Le et al. 2021) if each time series is viewed as a tree metric space with a root given by the starting point of the trajectory and under the choice of $p=2$.
- **Computational complexity:** The empirical $GW_\tau$ given by Definition 2 can be computed with linear complexity *O*(*N*) if trajectories have equal lengths ($N=n=m$) and with quadratic complexity $O(N^2)$ if lengths are unequal ($N=\max \{n,m\}$).

#### Proof of Proposition 1

- By Lemma 3.2 of Chowdhury and Mémoli (2019), we have that $GW_\tau$ between trajectories $(X,d_X,\mu _X)$ and $(Y,d_Y,\mu _Y)$ is a Wasserstein distance between measure pushforwards of $\mu _X$ and $\mu _Y$ under the functions $d_X(r_X,\cdot )$ and $\mu _Y(r_Y,\cdot )$, respectively, i.e.
  $$\begin{aligned} GW_\tau (X,Y) = \mathcal {W}_p(\left( d_X(r_X,\cdot )_\# \mu _X, d_Y(r_Y,\cdot )_\# \mu _Y \right) \end{aligned}$$
  Due to the metric properties of a Wasserstein distance (Theorem 7.3 of Villani (2021)), $GW_\tau$ defines a valid distance on $\mathcal {S}$. *Note* $GW_\tau$ will vanish for a pair of trajectories that have the same distribution of intrinsic distances from their starting points (see part (c) for the discussion of such distributions). Hence, two trajectories with the same distribution of distances from the start will be indistinguishable by $GW_\tau$. This is a common property of pseudo-distance constructions that only distinguish objects up to an equivalence class (Mémoli 2011), which is usually sufficient to distinguish the objects of interest in practical applications (see, for example, section 4 of Chowdhury and Mémoli 2019).
- The proof is provided in Appendix A. *Note* Denoting any of the lower bounds on GW from Mémoli (2011) by *LB*, the statement implies that $LB \le GW \le GW_\tau$. Thus, if *LB* is close to $GW_\tau$ for some pair of trajectories, the Gromov-Wasserstein distance can be approximated using the two bounds. In some of our empirical evaluations, we computed the *Third Lower Bound (TLB)* from Mémoli (2011) (using custom code that computes exact solution available in Supplementary Information) between pairs of trajectories of interest. Further, we numerically computed *GW* distance for the same pairs of trajectories using the function “$ot.gromov.gromov\_wasserstein2$” from Python Optimal Transport toolbox (Flamary et al. 2021) (code available in Supplementary Information). We observed that both *TLB* and *GW* produce trajectory comparison results similar to $GW_\tau$ (with $GW_\tau$ computed faster than both alternatives), with $TLB\le GW \le GW_\tau$ (data available in Supplementary Information).
- Consider distribution functions on $\mathbb {R}$ given by
  $$\begin{aligned} F(\xi ):= \mu _X\left( \{x \in X: d_X(r_X,x) \le \xi \}\right) \end{aligned}$$
  and
  $$\begin{aligned} G(\xi ):= \mu _Y\left( \{y \in Y: d_Y(r_Y,y) \le \xi \}\right) \end{aligned}$$
  and note that these functions represent a special case of *local distributions of distances* (Definition 5.5 of Mémoli (2011)) at points $r_X$ and $r_Y$, respectively. Observe further that the measures on $\mathbb {R}$ determined by *F* and *G* via assigning $F(b) - F(a)$ and $G(b) - G(a)$ to intervals [*a*, *b*] are precisely the pushforwards $d_X(r_X,\cdot )_\# \mu _X$ and $d_Y(r_Y,\cdot )_\# \mu _Y$. Comparing these pushforwards via Wasserstein distance $GW_\tau (X,Y) = \mathcal {W}_p(d_X(r_X,\cdot )_\# \mu _X,d_Y(r_Y,\cdot )_\# \mu _Y)$ can thus be interpreted as the comparison between local distributions of distances at the start of each trajectory.
- Consider the empirical case where trajectories $f = (X, d_X, \mu _X)$ and $g=(Y, d_Y, \mu _Y)$ are time series with finitely many points and with imposed discrete uniform measures as given in Definition 2. View *f* and *g* as trees with roots $r_X$ and $r_Y$, respectively. Note that the intrinsic polygonal arc length distance along each trajectory satisfies the definition of a tree metric given in Section 2.1 of Le et al. (2021), turning each trajectory into a *tree metric space*. For the choice $p=2$, the empirical $GW_\tau$ has the form
  $$\begin{aligned} \inf _{\mu \in \mathcal {C}(\mu _X, \mu _Y)} \left( \sum _{(x',y') \in X\times Y} \arrowvert d_X(r_X,x') - d_Y(r_Y,y') \arrowvert ^2 \, \mu (x',y') \right) ^{1/2} \end{aligned}$$
  which is the expression for *aligned-root FlowAlign* given in Le et al. (2021).
- By a well-known result in transportation theory (see, for example, Proposition 2.17 of Santambrogio 2015), since the measures $d_X(r_X, \cdot )_\# \mu _X$ and $d_Y(r_Y, \cdot )_\# \mu _Y$ are supported on $\mathbb {R}$ (namely, their supports are $d_X(r_X,\cdot )$ and $d_Y(r_Y,\cdot )$), the Wasserstein distance between them admits the closed form solution
  $$\begin{aligned} \mathcal {W}^p_p(\left( d_X(r_X,\cdot )_\# \mu _X, d_Y(r_Y,\cdot )_\# \mu _Y \right) = \int _0^1 \arrowvert F^{-1}(u) - G^{-1}(u) \arrowvert ^p \, du \end{aligned}$$
  where *F* and *G* are the distribution functions of measures $d_X(r_X,\cdot )_\# \mu _X$ and $d_Y(r_Y,\cdot )_\# \mu _Y$, respectively. For the empirical case, this integral is given by a formula involving sorted supports of the two measures (Remark 2.28 of Peyré et al. 2019). More precisely, if supports of the measures have the same sizes ($N=n=m$) and ordered as $z_1 \le \cdots , \le z_N$, $w_1 \le \cdots , \le w_N$, the Wasserstein distance of interest is given by
  11 $$\begin{aligned} \mathcal {W}^p_p(\left( d_X(r_X,\cdot )_\# \mu _X, d_Y(r_Y,\cdot )_\# \mu _Y \right) = \frac{1}{N} \sum _{i=1}^n \arrowvert z_i - w_i \arrowvert ^p \end{aligned}$$
  In general, it requires $O(N \log (N))$ operations to sort the vector of *N* support points (Section 5.1 of Knuth 1997), which is reported as a complexity of *align-root FlowAlign* in Le et al. (2021) (assuming *O*(1) complexity for computation of a single distance). In the specific case of time series, however, the supports come ordered after computation of the distances $d_X(r_X,\cdot )$ and $d_Y(r_Y,\cdot )$. Indeed, for a time series $\{f(t_i) \}_{i=1}^N$, computing the vector of distances $\{d_X(r_X,x_i) \}_{i=1}^N$ from the start of the trajectory requires $N-1$ successive additions of the form $d_X(r_X,x_i) + d_X(x_i,x_{i+1})$. This results in *O*(*N*) complexity for the sorted supports, followed by *O*(*N*) complexity of computing the summation in Eq. (11), giving the total *O*(*N*) complexity for the $N=n=m$ case. If supports have unequal sizes ($n \ne m$), the expression has the form
  12 $$\begin{aligned} \mathcal {W}^p_p(\left( d_X(r_X,\cdot )_\# \mu _X, d_Y(r_Y,\cdot )_\# \mu _Y \right) = \sum _{i=1}^n \sum _{j=1}^m \lambda _{ij} \arrowvert z_i - w_j \arrowvert ^p \end{aligned}$$
  where $\lambda _{ij} = \left( \frac{i}{n}\wedge \frac{j}{m} - \frac{i-1}{n}\vee \frac{j-1}{m} \right) \cdot \chi _{\{im \wedge jn > (i-1)m \vee (j-1)n\}}$ (Weitkamp et al. 2022). In this case, the complexity of the double summation dominates, giving an overall asymptotic complexity of $O(N^2)$. $\square$

#### Example 1

($\mathbf {GW_\tau }$ **between trajectories**) Consider the four trajectories shown in Fig. 1B, corresponding to the following shapes: two straight line (3D and 2D, red lines) and two circular shapes (3D and 2D, green lines). The lines are defined on discrete time points unequally spaced in [0, 2], and circular shapes are defined on discrete time points unequally spaced in $[0,23\pi /12]$. Individual time points are indicated by black dots on all trajectories. The exact functional forms underlying these trajectories are:

$$\begin{aligned} f_1(t):= & {} \frac{1}{\sqrt{2}}(\cos t, \sin t, t) \text { 3D ``circular'' shape (helix) } \\ f_2(t):= & {} (\cos t, \sin t) \text { 2D circle} \\ f_3(t):= & {} \frac{1}{\sqrt{3}} (t, t, t) \text { 3D line} \\ f_4(t):= & {} \frac{1}{\sqrt{2}} (t, t) \text { 2D line} \end{aligned}$$

For each of the four trajectories $f \in \{f_i\} _{i=1}^4$ of length $n_i$, we compute the vector $v_k$ of intrinsic distances from the start of each trajectory $r_X=f(t_1)$ to a point $x_k = f(t_k)$, $k=1,\cdots , n_i$ whose *k*th entry is, according to Eq. (8),

$$\begin{aligned} d_X(r_X,x_k) = \Vert x_2 - r_X \Vert + \Vert x_3 - x_2 \Vert + \ldots + \Vert x_k-x_{k-1} \Vert \end{aligned}$$

(This is done by the function “vec$\_$geo$\_$dist.m” in the software provided for this paper, see Supplementary Information).

To find $GW_\tau (f_i,f_j)$, we take the vectors of intrinsic distances $v(f_i)$ and $v(f_j)$ as computed above, and use the closed form expression from the proof of Proposition 1e (Eq. 12) giving

$$\begin{aligned} GW_\tau ^p(f_i,f_j) = \sum _{l=1}^{n_i} \sum _{r=1}^{n_j} \lambda _{lr} \arrowvert z_l - w_r \arrowvert ^p \end{aligned}$$

where $\lambda _{lr} = \left( \frac{l}{n_i}\wedge \frac{r}{n_j} - \frac{l-1}{n_i}\vee \frac{r-1}{n_j} \right) \cdot \chi _{\{ln_j \wedge rn_i > (l-1)n_j \vee (r-1)n_i\}}$. The values of $\{z_l \}$ and $\{w_r \}$ are precisely the entries of $v(f_i)$ and $v(f_j)$ sorted in descending order. If the trajectories had the same number of points $n_i=n_j=N$, the computation would be even simpler (Eq. 11)

$$\begin{aligned} GW_\tau ^p(f_i,f_j) = \frac{1}{N} \sum _{l=1}^N \arrowvert z_l - w_l \arrowvert ^p \end{aligned}$$

Taking the *p*th root gives the value $GW_\tau (f_i,f_j)$ for the $4 \times 4$ matrix of $GW_\tau$ pairwise distances between trajectories. Each entry $GW_\tau (f_i,f_j)$ of this matrix is computed by the function “wass$\_$sorted.m” in the software provided for this paper (see Supplementary Information).

Note that the matrix of $GW_\tau$ distances is symmetric due to the symmetry of the Wasserstein distance, and hence only the upper portion needs to be computed.

#### Remark 1

(**Practical note on computation**) As Example 1 illustrates, for real-world data applications, the empirical $GW_\tau$ given by Definition 2 is computed according to the closed-form expression (11) (for trajectories of equal length) or (12) (for trajectories of unequal lengths), which are both faster and easier than using the definition directly.

## Results and Discussion

We illustrate the performance of $GW_\tau$ on various supervised and unsupervised machine learning tasks using biologically relevant time series datasets with diverse characteristics. We empirically demonstrate that $GW_\tau$ is able to distinguish trajectories that are known to belong to different classes more accurately and/or efficiently than other commonly used distance measures. We also discuss how and why each case illustrates our more general claim that $GW_\tau$ is useful for biological time series comparison.

While $GW_\tau$ can potentially be used to compare trajectories that lie in spaces of different dimensions (see Fig. 1B and Example 1), real-world data applications often call for a comparison of data from spaces with the same dimensionality. This case is our focus in this section. We compare $GW_\tau$ with the most frequently used Euclidean and Dynamic Time Warping (DTW) distances (Dau et al. 2018; Abanda et al. 2019), with $p=2$ as the exponent for the Euclidean distance, and unconstrained DTW as computed by the Matlab function *dtw*. The $GW_\tau$ distance with exponent $p=2$ is given by Definition 2 and is computed using Proposition 1(a), (e) as a Wasserstein distance with closed-from expression (Eq. 11 for trajectories of equal sizes, or Eq. 12 for trajectories of unequal sizes). For all applications, we use the workflow outlined in Fig. 1C.

**Fig. 2:**
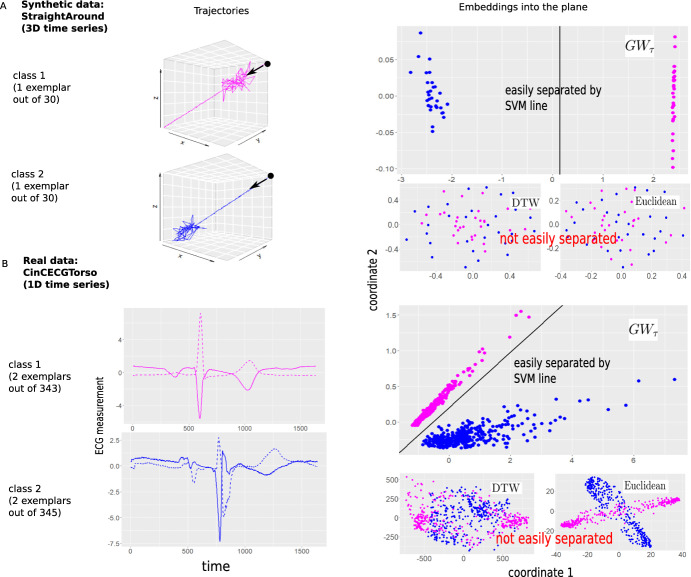
$GW_\tau$ performance in embedding and subsequent separation of classes in comparison to dynamic time warping (DTW) and Euclidean distances: synthetic and real world data (synthetic data is constructed to resemble real data characteristics in higher dimensions). The $GW_\tau$ distance matrix is used to embed time series from synthetic (**A**) and real (**B**) data (Left panel) into the plane, allowing for accurate separation of classes (right panel). It is more difficult to separate classes when embedding is performed with DTW or Euclidean distances in comparison to $GW_\tau$ distance (Color figure online)

**Fig. 3:**
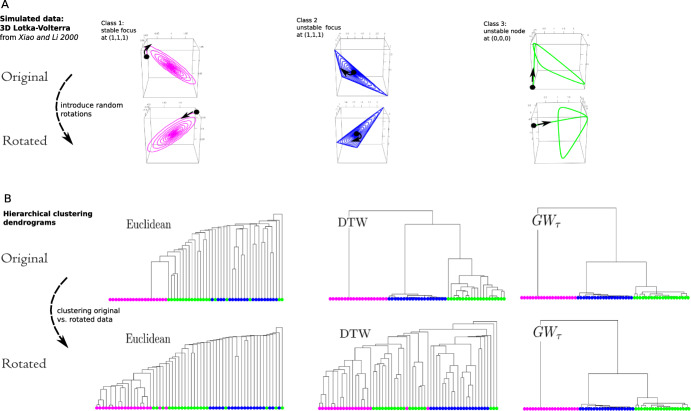
$GW_\tau$ performance in hierarchical clustering in comparison to Euclidean and dynamic time warping (DTW) distances: model simulation data. **A** top: simulated data from the three dimensional Lotka–Volterra system from Xiao and Li (2000). Three classes correspond to solution trajectories when starting in proximity to a stable focus (1, 1, 1) (Class 1), an unstable focus (1, 1, 1) (Class 2) or an unstable node (0, 0, 0) (Class 3), with 20 trajectories in each class corresponding to random initial conditions (one trajectory from each class is shown). Bottom: randomly rotated data (one trajectory from each is shown). **B** hierarchical (single linkage) clustering dendrograms constructed using Euclidean, DTW, and $GW_\tau$ distances as dissimilarity measures between trajectories for original data (top) and “rotation-corrupted” data (bottom). Note poor performance of Euclidean distance in both cases, and rapid decrease in performance of DTW distance when rotational noise is introduced. The performance of $GW_\tau$ is high in both cases (Color figure online)

**Fig. 4:**
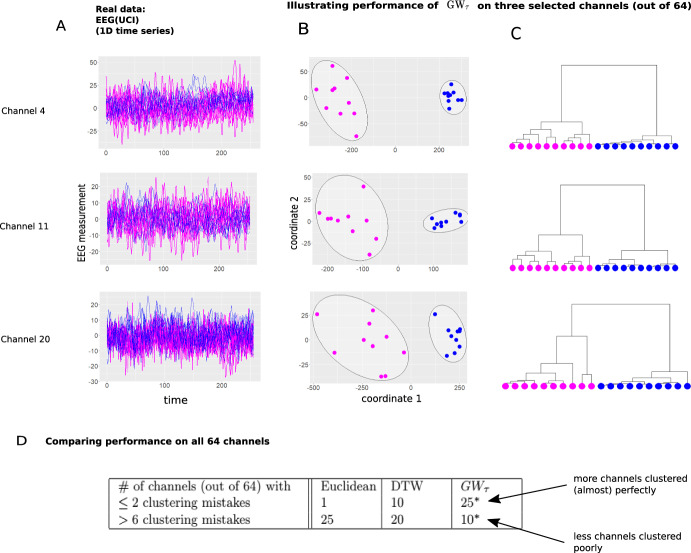
$GW_\tau$ performance on embedding and clustering: real world data. **A** electroencephalogram (EEG) data on selected three EEG channels (out of total 64 available in the dataset; Dua and Graff (2017)): 10 trajectories in each class represent EEG response to a stimulus for an alcoholic (magenta, Class 1) vs. non-alcoholic (blue, Class 2) patient. **B** Embeddings of the data into the plane using $GW_\tau$ distance matrices and results of k-means clustering in the embedded space. **C** hierarchical cluster (complete linkage) dendrograms using $GW_\tau$ distance matrices. **D** k-means clustering results on embedded data (such as in panel B): reporting number of channels (out of 64 total) with small ($\le 2$) and large ($>6$) number of incorrectly clustered trajectories (“clustering mistakes”) when using Euclidean, DTW, and $GW_\tau$ distances. Note superior performance of $GW_\tau$ in this comprehensive evaluation (Color figure online)

**Fig. 5:**
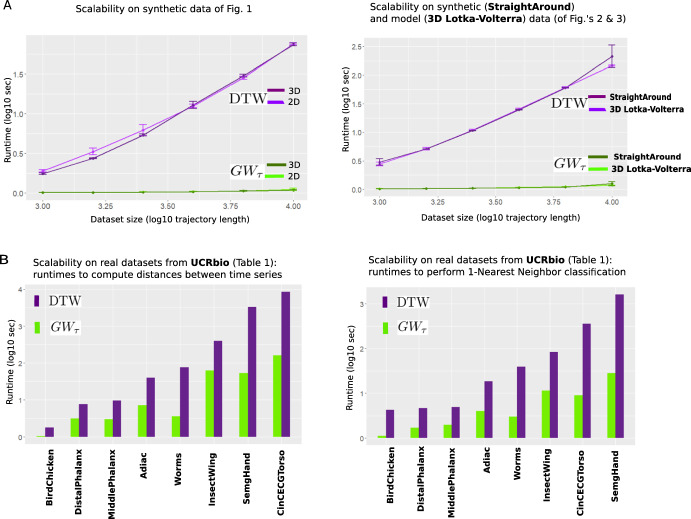
Scalability of $GW\tau$ with respect to dataset size and dimension. **A** left: runtimes (log scale) when calculating distances for 100 circle/line pairs (synthetic data used in Fig. 1B) in both 2D and 3D using $GW_\tau$ versus DTW. As expected, increase in dimension from 2D to 3D does not affect the runtimes; increase in dataset size (as number of points along each trajectory) results in steep increase in runtimes for DTW, while has almost negligible effect on $GW_\tau$. The same trend is observed for the other two synthetic/simulated datasets used in Fig. 2A and 3 (right). **B**. runtimes (log scale) when calculating matrices of all pairwise distances between trajectories (left) and performing 1-NN classification (right) using $GW_\tau$ versus DTW for the real data **UCRbio** used in Table 1, listed in increasing data complexity appropriate for each task (t.s.length*(train size + test size) (left) and t.s.length*train size* test size (right)). Observe shorter runtimes when using $GW_\tau$ compared to DTW (Color figure online)

**Fig. 6:**
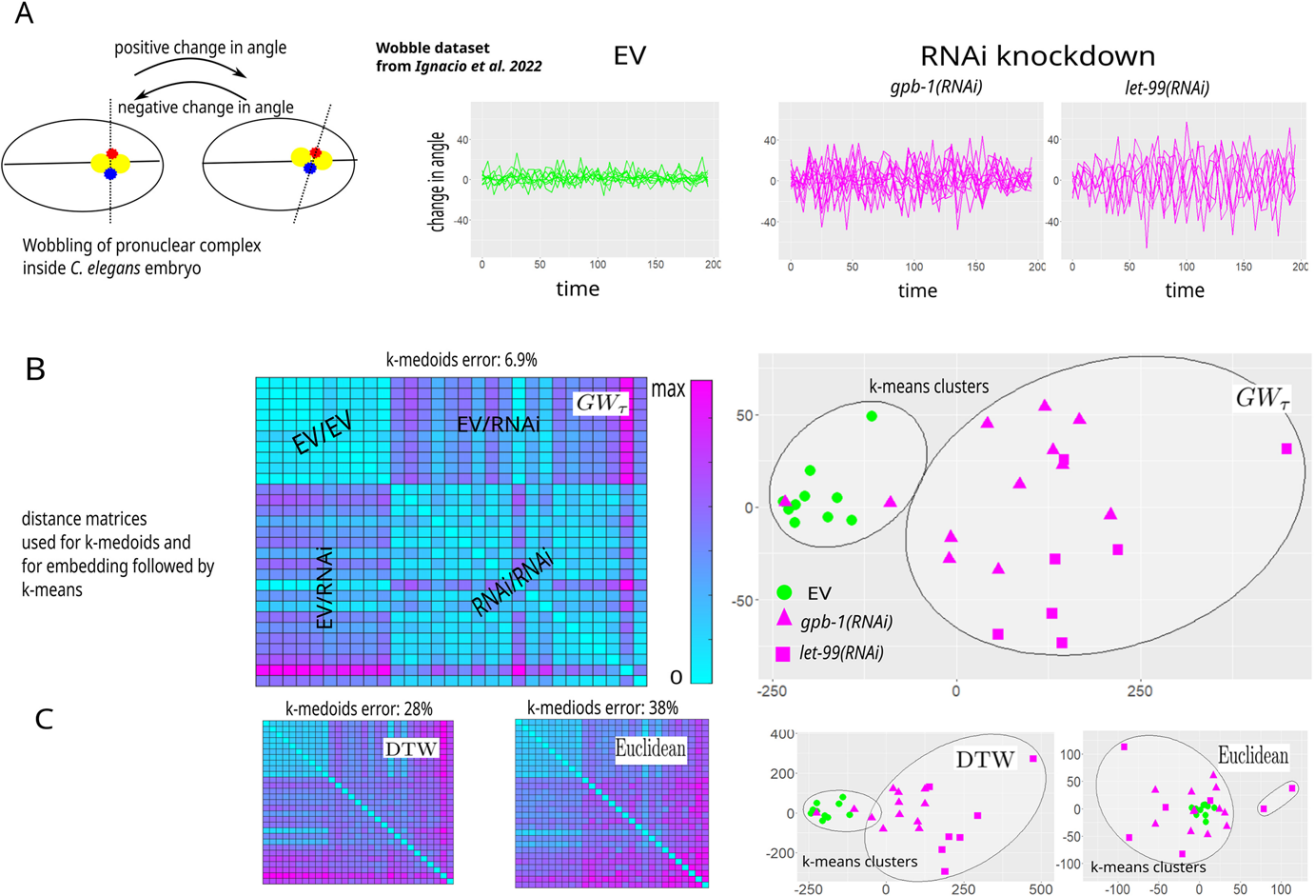
Applying $GW_\tau$ for the analysis of time series arising in cell biology: grouping trajectories from cells under different experimental conditions. **A** schematic of the “wobbling” movement quantification and corresponding **Wobble** dataset from Ignacio et al. (2022) for change in angle of a pronuclear complex (yellow, with two centrosomes marked by red and blue) during centration and rotation in early *C. elegans* embryos (10 trajectories of empty vector (EV) control (Left), 12 trajectories of cells subjected to RNA interference against the protein GPB-1 (*gpb-1(RNAi)*; Center), and 7 trajectories of cells subjected to RNA interference against the protein LET-99 (*let-99(RNAi)*; Right)). **B** using $GW_\tau$ to construct the distance matrix between trajectories (left) to be used for k-medoids clustering and embedding of trajectories into the plane followed by k-means clustering on embedded coordinates. Both clustering methods using $GW_\tau$ distinguish EV from the RNAi knockdowns, with two RNAi knockdown trajectories found closer to EV than to other RNAi knockdown trajectories. **C** DTW and Euclidean distances have larger error in distinguishing EV from the RNAi knockdowns (Color figure online)

**Fig. 7:**
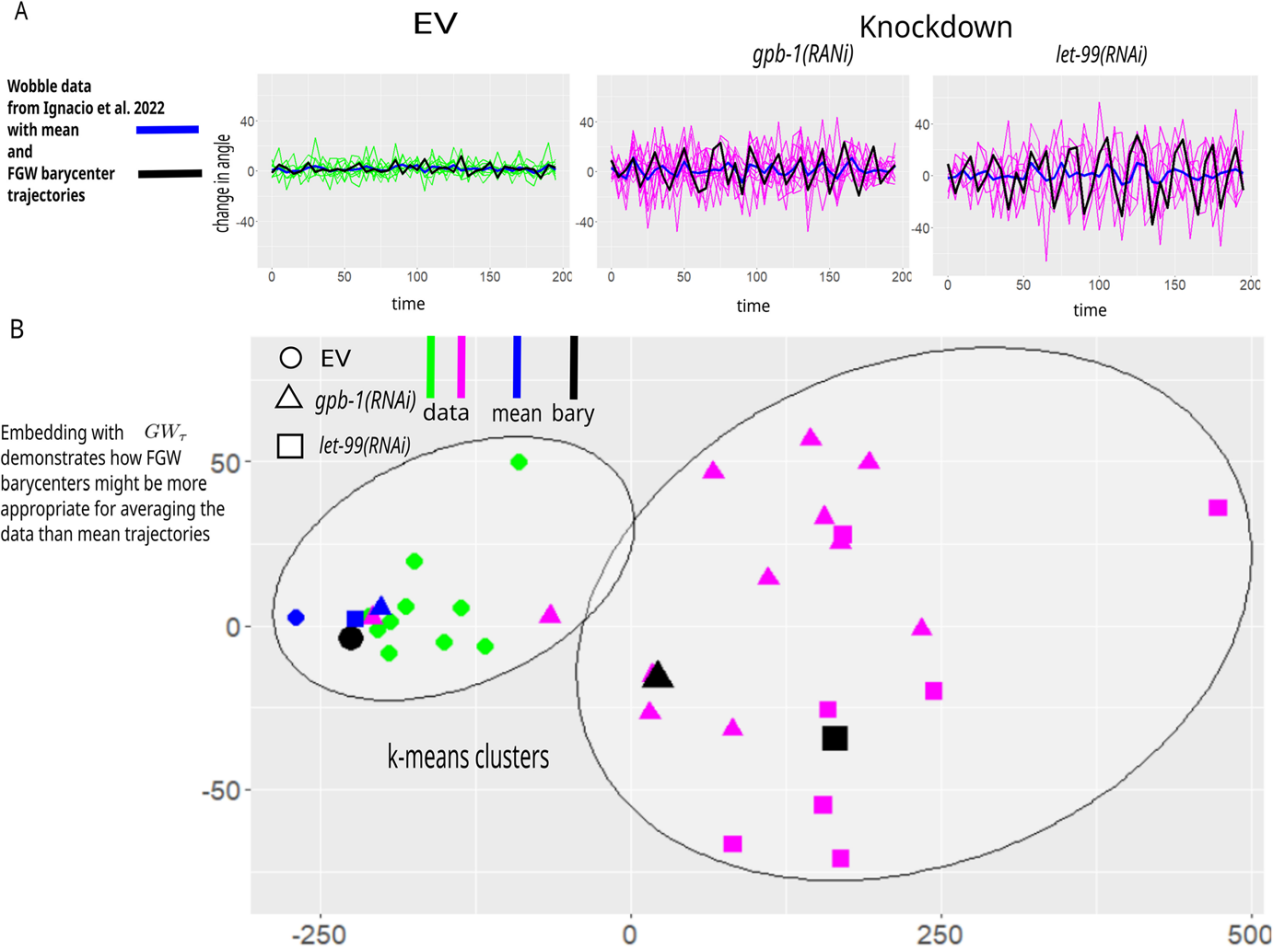
Applying $GW_\tau$ to analyse time series arising in cell biology: comparing different trajectory averaging methods. **A** **Wobble** dataset from Ignacio et al. (2022) with mean trajectories and FGW barycenter trajectories based on FGW barycenter method of Vayer et al. (2020). Note that traditionally used mean trajectories appear to damp the oscillations found in the RNAi treatment data. **B** embedding with $GW_\tau$ places mean trajectories of the RNAi-treated embryos (blue) inside the EV group, while the FGW barycenter trajectories (black) stay close to their respective trajectories (Color figure online)

The results of our empirical evaluations are summarized in Table 1 and Figs. 2, 3, 4, 5, 6 and 7. Table 1 focuses on eight biologically relevant datasets from the UCR Time Series Classification Archive (Dau et al. 2018), named **UCRbio** in what follows. Figures 2, 3 and 4 are concerned with synthetic data (which we name **StraightAround**; Fig. 2), simulated data from a biological model (**3D Lotka-Volterra** system from Xiao and Li (2000); Fig. 3), and two biologically relevant publicly available datasets composed of physical data: (**CinCECGTorso**, and the dataset that we name **EEG(UCI)**; Fig. 4). Figure 5 illustrates how runtimes of our method scale with data complexity (scalability results). Figures 6 and 7 present an analysis of pronuclear movement data from the early embryo of the nematode worm *Caenorhabditis elegans* (Ignacio et al. 2022; we name this dataset **Wobble**). Below we describe each dataset, its biological relevance, and show how $GW_\tau$ outperforms Euclidean and DTW when used for various clustering and/or classification tasks.

### Empirical Results on Synthetic, Model-Based, and Publicly Available Real-World Data

**Table 1:** Performance of $GW_\tau$ on 1-Nearest Neighbor classification task on selected biologically relevant datasets from UCR Time Series Classification Archive ( Dau et al. (2018); **UCRbio**)

UCR dataset name	$\#$ classes	$\text {t.s.}$ length	train size	test size	Euclidean error	$GW_\tau$ error	DTW error
CinCECGTorso	4	1639	40	1380	0.1029	$\mathbf{0.1290}^*$	**0.3493**
InsectWingbeatSound	11	265	220	1980	0.4384	$\mathbf{0.5995}^*$	**0.6449**
DistalPhalanxOutlineAgeGroup	3	80	400	139	**0.3741**	$\mathbf{0.3237}^*$	0.2302
Worms	5	900	181	77	**0.5455**	$\mathbf{0.5325}^*$	0.4156
Adiac	37	176	390	391	**0.3887**	$$\mathbf{0.3555}^{**}$$	**0.3964**
BirdChicken	2	512	20	20	**0.4500**	$$\mathbf{0.1500}^{**}$$	**0.2500**
MiddlePhalanxOutlineAgeGroup	3	80	400	154	**0.4805**	$$\mathbf{0.4740}^{**}$$	**0.5000**
SemgHandMovementCh2	6	1500	450	450	**0.6311**	$$\mathbf{0.3933}^{**}$$	**0.4156**

Train and test sizes indicate the number of trajectories in each set and t.s. length (time series length) indicates the number of points along each trajectory in a given dataset, as chosen by UCR. Bold indicate instances when $GW_\tau$ outperforms either Dynamic Time Warping (DTW, unconstrained, data taken from Dau et al. (2018), column denoted *DTW(w=0)*) or Euclidean distance (single star), or both (double star)

*UCRbio (Table* 1) and general discussion of DTW and Euclidean comparison As a first result, we report the performance of our distance $GW_\tau$ in the classical machine learning task of 1-Nearest Neighbor (1-NN) classification (the description can be found, for example, in Hastie et al. 2009). We discuss the general features when comparing these time series using DTW and Euclidean distance. For this comparison, we consider eight biologically relevant datasets from the UCR Time Series Classification Archive (Dau et al. 2018) with diverse characteristics such as number of classes, sizes of training and testing sets, lengths of time series, and, most importantly, the wide range of trajectory behaviors. The time series in these selected datasets represent different types of biologically relevant data, including electrocardiogram measurements for different cardiology patients (*CinCECGTorso* dataset), power spectra of insect sounds for different classes of insects (*InsectWingbeatSound*), or image data on different types of unicellular algae (*Adiac*).

The goal of classification is to construct a classifier based on the training data with known class labels (the training set contains several trajectories from each class) that can accurately predict the class of a given sample from the test data. A classifier is constructed using pairwise distances between time series, and thus the performance of any particular classifier is determined by the underlying distance between time series. As suggested by Dau et al. (2018), we fix the classifier type to 1-NN and report how a proposed distance performs in this task. While many successful distance measures are proposed in the literature, no single distance is expected to outperform others on all datasets; it is noted, however, that Euclidean and DTW distances show very strong performance on most datasets (Dau et al. 2018). We thus report the results comparing $GW_\tau$ to these two distances (Table 1).

The role of a time series distance in 1-NN classification, as well as many other classification and clustering tasks, is to capture the features of time series that are indicative of a class label. For an unsupervised clustering task, these labels are not known but are hoped to be meaningfully inferred (Hastie et al. 2009). When time series in different classes have similar behavior up to a shift, it would likely be difficult for the DTW to distinguish these classes, as DTW aligns regions with similar types of behavior (see further discussion on this for the **StraightAround** and **CinCECGTorso** cases). However, $GW_\tau$ is able to distinguish between shifts since a shifted version of a trajectory has a different distribution of intrinsic distances, and the larger the shift, the easier it is for $GW_\tau$ to distinguish between the trajectories, causing $GW_\tau$ to outperform DTW (rows 1–2 and 5–8 of Table 1). As time shifts in activity often serve as important markers of certain biological behavior (Liu et al. 2010) and the identification of significant time points along trajectories plays a crucial role in mathematical modeling of many time-dependent biological processes (McGee and Buzzard 2018), successfully capturing time shifts as class indicators is a desirable property of an algorithm that compares biological time series.

Another situation common to biological time series arises when time series within the same class have similar qualitative behavior, for example, oscillations (Kruse and Jülicher 2005), but due to slight phase shifts within the class, the time series appear different and maybe even similar to an opposite class with different characteristics with respect to features such as oscillation frequency and/or amplitude (see more on this in the discussion of the **3D Lotka-Volterra** data). In these cases, Euclidean distance may not capture the features responsible for a class label. On the other hand, $GW_\tau$ performs well in such cases by looking at the internal distances from the start of each trajectory. Slightly shifted versions of qualitatively similar trajectories will be matched by an optimal transport routine producing a small distance between such trajectories. At the same time, qualitatively different trajectories will have higher values of $GW_\tau$ since it is more difficult to match trajectories when their internal distances are very different. This causes $GW_\tau$ to outperform Euclidean distance in 1-NN classification in these cases (Table 1, rows 3–8).

Next, we demonstrate that $GW_\tau$ outperforms both DTW and Euclidean distances for other biological time series datasets when considering common machine learning problems such as grouping and clustering. As shown in Fig. 1C, clustering can be distance-based and performed directly on the distance matrix; two such clustering methods are considered, the k-medoids and hierarchical clustering (Hastie et al. 2009). Another approach is to first embed the trajectories into the plane via Multidimensional Scaling (MDS, see Hastie et al. (2009) for description) using a distance matrix of interest, and then perform clustering using coordinates of the resulting embedding (as outlined in Fig. 1C); here we consider k-means clustering of embedded points. The datasets we chose for this evaluation have a known class label attached to each trajectory, either specified by a field expert in the real world data case (for the datasets **CinCECGTroso**, **EEG(UCI)**, and **Wobble** dataset of Sect. 3.2) or imposed during construction of the data in synthetic (**StraightAround**) and model-based (**3D Lotka-Volterra**) cases. For all unsupervised clustering tasks, we pretend to be unaware of these class labels when grouping the points, and we only apply class labels after clustering to assess the quality of clustering. We emphasize our interest in performance of a *distance* with a (fixed) clustering algorithm rather than performance of a clustering algorithm given the distance; hence, we are not searching for the best clustering algorithm in each case, but rather using the most common ones to demonstrate how switching from DTW or Euclidean distance to $GW_\tau$ can improve the results under a given clustering procedure.

StraightAround (Fig. 2A) is a synthetic dataset of 3D time series with two types of behavior (2 classes): starting from the point (1, 1, 1), each trajectory follows a straight line path to the origin with small Gaussian noise, and some random (Gaussian with higher variance) excursion either toward the beginning (Class 1) or the end (Class 2) of the path (30 trajectories in each class defined on a common set of time instances of length $n=m=300$ points). The goal of constructing this data was to illustrate (in 3D) that $GW_{\tau }$ can be used to correctly classify time series datasets when a shift of activity is indicative of a class label.

CinCECGTorso (Fig. 2B), a two-class version of a **UCRbio** dataset. Here we consider a test set from UCR dataset *CinCECGTorso* (Dau et al. 2018), where we chose two classes (3 and 4 from the original dataset) out of a total of four available. Each class contains electrocardiogram (ECG) measurements that constitute one heatbeat for the same patient (343 heatbeats for Class 1 and 345 heatbeats for Class 2, all trajectories having length $n = m = 1639$).

As noticed previously in the literature Lubba et al. (2019), the important feature distinguishing the classes is the slight difference in timing of the peak. This time shift allows Euclidean distance to outperform both DTW and $GW_\tau$ in 1-NN classification on the full four-class **UCRbio** version. However, for the two-class version of the data, the Euclidean distance performs weaker in the embedding task than $GW_\tau$ (with DTW still remaining a weaker alternative).

#### Remark 2

For both synthetic and real data of Fig. 2, we plot the linear SVM classifier (Hastie et al. 2009) for the embedded points merely to illustrate that they can be easily linearly separated. We do not use this constructed classifier for any further classification, and we leave investigation of its performance for future study.

3D Lotka-Volterra (Figure 3) is a simulated dataset based on the model from Xiao and Li (2000). The model is a specific case of a three-dimensional Lotka-Volterra system constructed to illustrate the bifurcation dynamics leading to limit cycles in different parameter regimes (model equations are given in Appendix B). Here we consider three parameter regimes that give rise to our three classes of trajectories corresponding to starting in a proximity to the following steady states: stable focus at (1, 1, 1) (Class 1), unstable focus at (1, 1, 1) (Class 2), and unstable node at (0, 0, 0) (class 3), with 20 trajectories in each class corresponding to randomly sampled initial conditions (each trajectory is $n=m=1000$ points in length).

We now pretend to be unaware of class labels and consider an unsupervised task of clustering the trajectories based on the distance matrices given by $GW_\tau$, DTW, and Euclidean distances between trajectories. We perform hierarchical (single linkage) clustering (see Hastie et al. (2009) for the description) of trajectories with dissimilarity between trajectories given by Euclidean, DTW, and $GW_\tau$ distances. Further, we subject the data to random rotations in 3D space and demonstrate strong performance of $GW_\tau$ for both unperturbed and perturbed data, in contrast to Euclidean and DTW distances.

EEG(UCI) (Fig. 4) Our last summary result concerns the publicly available dataset from the UCI machine learning repository (Dua and Graff 2017). We used the dataset coded as *smni97*$\_$eeg$\_$*data.tar.gz* that can be downloaded following the link https://archive.ics.uci.edu/ml/datasets/eeg+database. The dataset provides electroencephalogram (EEG) measurements for two patients: one diagnosed with alcoholism (class 1) and one control (class 2). Data on 10 time series corresponding to 10 repetitions of the experiment is available for each class, with 64 different channels corresponding to 64 electrodes (non-invasively) attached to a patients’ scalp (each time series has length $n=m=256$).

We again pretend to be unaware of class labels, and we subject the data to an unsupervised task of clustering the trajectories in each of the 64 channels into two clusters, hoping that each cluster would contain 10 trajectories corresponding to the same patient. Similar to previous examples, $GW_\tau$ provides distance matrices that allow for meaningful clustering, either using the distance matrix directly in the hierarchical clustering case, or embedding the data into the plane via MDS and subjecting the embedded data to k-means using the 2D coordinates of the embedding.

Scalability (Fig. 5) Here we demonstrate that $GW_\tau$ distance not only captures similarities/differences that Euclidean and DTW distances have difficulties capturing, but also that $GW_\tau$ is indeed fast to compute in comparison to DTW. Since Euclidean distance is essentially a subroutine for both $GW_\tau$ and DTW, it is of course faster (but as above results suggest, by itself it is not always possible/useful) to compute, and hence we omit it from our comparisons in this section.

Theoretical complexity of single $GW_\tau$ computation for all the datasets of Fig. 5 is *O*(*N*) (Proposition 1e), while theoretical complexity of single computation of classical is $O(N^2)$ Keogh and Ratanamahatana (2005) (where *N* is the common trajectory length in each dataset), and thus algorithms using $GW_\tau$ are expected to run faster than the ones using classical DTW. To confirm this empirically, we report^7^ runtimes for several tasks performed in this paper using $GW_\tau$ and DTW on the datasets whose dimension/size we can control (Fig. 5A) as well as on the real-world datasets whose (mostly large) complexity cannot be changed (Fig. 5B).

### Using $GW_\tau$ for Analysis of **Wobble** Dataset from Ignacio et al. (2022)

Our final result demonstrates how our $GW_\tau$ distance is used to investigate differences in trajectory behavior and compare averaging methods for data arising in cell biology. The data recently published in Ignacio et al. (2022) investigated the effect of three different experimental conditions on pronuclear movement in early embryos of the nematode worm *Caenorhabditis elegans (C. elegans)*: empty vector (EV) as the control treatment, RNA interference (RNAi) to knockdown the protein GPB-1 (*gpb-1(RNAi)*), and RNAi to knockdown the protein LET-99 (*let-99(RNAi)*). It was observed that compared to EV embryos, embryos subjected to RNAi knockdown exhibit a pronuclear movement defect termed *wobble* in Ignacio et al. (2022), which is characterized by oscillations of the pronuclear complex, and quantified by the change in angle between the centrosome axis and the long axis of the embryo (Fig. 6A). The **Wobble** dataset corresponding to this data consists of trajectories for the change in angle over time, with 10 trajectories for the EV condition, 12 trajectories for *gpb-1(RNAi)*, and 7 trajectories for *let-99(RNAi)*. All trajectories are defined on the same time vector of 40 time points, with equally spaced 5 s intervals (Fig. 6A).

The data analysis in Ignacio et al. (2022) employs the discrete Fourier transform to confirm the observation that the behavior of the RNAi knockdown embryos indeed exhibit wobbling, while the EV control embryos do not. Remarkably, this result is confirmed with a completely different type of analysis when clustering the dataset using $GW_\tau$ as a distance between trajectories (Fig. 6B). Although not entirely unreasonable, DTW distance performs slightly weaker in reproducing EV/RNAi clusters, and the Euclidean distance performance is rather unsatisfactory (Fig. 6C).

This result suggests that averaging trajectories within EV or RNAi knockdown groups, respectively, using Euclidean distance may not accurately preserve the features of individual trajectories in the mean trajectory. Rather, other averaging methods may perform better for datasets of this type when Euclidean distance is not capturing similarities within the class and differences between classes. Among other plausible alternatives, the Fused Gromov-Wasserstein (FGW) is the barycentering method recently proposed to average trajectories under the Gromov-Wasserstein framework (Vayer et al. 2020).^8^ We observe that FGW barycenters (computed using the function $ot.gromov.fgw\_barycenters$ from Python Optimal Transport toolbox (Flamary et al. 2021) with parameter $\alpha =0.5$^9^) provide a plausible solution to the averaging problem for these data (Fig. 7A). Interestingly, FGW barycenters are close to the individual datasets in the $GW_\tau$ sense (Fig. 7B), even though the distance used in the FGW barycenter problem has little in common with $GW_\tau$ except the overall conceptual Gromov-Wasserstein paradigm (see section of Vayer et al. (2020) for the definition of FGW distance and comparison with other GW-type constructions). Hence, closeness in the $GW_\tau$ sense is not an artifact of barycenter construction, but rather evidence that FGW barycenter trajectories are similar in shape to the data from their corresponding experimental conditions. In contrast, the mean trajectories (which are, in fact, the barycenters under 1D Euclidean distance between *y*-coordinates) for *gpb-1(RNAi)* and *let-99(RNAi)* are closer to the EV group than to their corresponding RNAi groups in terms of the shapes, as $GW_\tau$ comparison shows (Fig. 6B). This result illustrates how $GW_\tau$ can assess the quality of an averaging procedure for biological time series.

To summarize the findings of this paper, we introduced a distance between time series that we termed $GW_\tau$ and demonstrated its performance for comparison of biological time series. The construction $GW_\tau$ is based on fixing two coordinates in the Gromov-Wasserstein distance optimization program, which turns the resulting problem into a Wasserstein distance optimization program on the real line. Having a closed-form solution, this program is scalable in terms of the number of points along a given time series, providing a quick and exact alternative to other time series distances. Various empirical evaluations on synthetic and real world datasets demonstrate the use of our proposed distance for biological time series comparison and further applications.

## Supplementary information

**Data and code** The data used in this paper and the codes to reproduce numerical results, along with sample tutorial on computing $GW_\tau$ distance, are available at https://github.com/kravtsova2/GWtau.

## Appendix A Proof of Proposition 1(b)

The proof is given for the continuous case. The proof for the discrete case is the same with summation replacing integration.

Let $p \ge 1$, and recall that for all $a,b \in \mathbb {R}$, $\arrowvert a + b \arrowvert ^p \le 2^{p-1}(\arrowvert a \arrowvert ^p + \arrowvert b \arrowvert ^p)$. Suppose WLOG that the point $x'$ is “farther" from the start of the curve than the point *x*, i.e. $d_X(r_X,x') > d_X(r_X,x)$. Then, we can write $d_X(x,x') = d_X(r_X,x') - d_X(r_X,x)$, and thus for any $\mu \in \mathcal {C}(\mu _X, \mu _Y)$,

$$\begin{aligned}{} & {} \int _{X \times Y} \int _{X \times Y} \arrowvert d_X(x,x') - d_Y(y,y')\arrowvert ^p \, d\mu (x',y') d\mu (x,y) \\{} & {} \quad = \int _{X \times Y} \int _{X \times Y} \arrowvert ( d_X(r_X,x')\\{} & {} \qquad - d_X(r_X,x) ) - (d_Y(r_Y,y') - d_Y(r_Y,y))\arrowvert ^p \, d\mu (x',y') d\mu (x,y) \\{} & {} \quad = \int _{X \times Y} \int _{X \times Y} \arrowvert ( d_X(r_X,x') - d_Y(r_Y,y') ) - (d_X(r_X,x) - d_Y(r_Y,y))\arrowvert ^p \,\\{} & {} \qquad \quad d\mu (x',y') d\mu (x,y) \\{} & {} \quad \le 2^{p-1} \cdot \Bigl (\int _{X \times Y} \int _{X \times Y} \arrowvert d_X(r_X,x') \\{} & {} \qquad - d_Y(r_Y,y') \arrowvert ^p \, d\mu (x',y') d\mu (x,y) \\{} & {} \qquad + \int _{X \times Y} \int _{X \times Y} \arrowvert (d_X(r_X,x) - d_Y(r_Y,y) \arrowvert ^p \, d\mu (x',y') d\mu (x,y) \Bigr ) \\{} & {} \quad = 2^{p-1} \cdot 2 \int _{X \times Y} \arrowvert d_X(r_X,x) - d_Y(r_Y,y)\arrowvert ^p \, d\mu (x,y) \end{aligned}$$

The last equality follows since one of the two integrals in each term integrates a constant function with respect to a probability measure $\mu$ resulting in a value of 1. Taking infimum over $\mu \in \mathcal {C}(\mu _X, \mu _Y)$ gives

$$\begin{aligned} (2 GW(X,Y))^p \le 2^p (GW_\tau (X, Y))^p \end{aligned}$$

and dividing by $2^p$ and taking the *p*th root gives that $GW \le GW_\tau$, as claimed.

## Appendix B Model equations constructed by Xiao and Li (2000) and used to produce the dataset **3D Lotka-Volterra**

Denote the parameter vector $\varepsilon := (\varepsilon _1, \varepsilon _2)$. For $0<\Vert \varepsilon \Vert \ll 1$, consider the system given in Equation (6) of Xiao and Li (2000) as

B1 $$\begin{aligned} \dot{x}_1&=x_1[(1-x_1)+(1-x_2)+(1-x_3)] \end{aligned}$$

B2 $$\begin{aligned} \dot{x}_2&=x_2[(1-x_1)+(1-x_2)+2(1-x_3)] \end{aligned}$$

B3 $$\begin{aligned} \dot{x}_3&=x_3[(\frac{13}{5}+\varepsilon _1)(1-x_1)+(\frac{8}{5}+\varepsilon _2)(1-x_2)+3(1-x_3)] \end{aligned}$$

According to the analysis of pp. 9–11 of Xiao and Li (2000), the following parameter regimes correspond to the following stability results for the equillibrium points:

- When $3\varepsilon _2 + 2\varepsilon _1>0$, the equilibrium (1, 1, 1) is an unstable focus
- When $3\varepsilon _2+2\varepsilon _1<0$, the equilibrium (1, 1, 1) is a stable focus
- When $(\varepsilon _1,\varepsilon _2,) = (0,0)$, the equilibrium (0, 0, 0) is an unstable node

These three types of behavior constitute the three classes in our dataset **3D Lotka-Volterra** (Fig. 3).

## Footnotes

1 Following common practice, we use the term “distance” for any dissimilarity measure between two trajectories, even if the measure fails to satisfy some aspects of the mathematical definition of distance.

2 The map is usually termed *(parameterized) curve* in mathematical literature (for example, Do Carmo (2016)).

3 These assumptions ensure that the trajectory is a compact Polish space which is needed for further discussion.

4 Under this specification, the time coordinate does not explicitly appear in the last two cases, unlike the first case; we observed that this choice provides the most computationally efficient results.

5 Any given time series, regardless of underlying *f* (if any), has finitely many points, and hence it is compact and Polish.

6 Similarly to the continuous case, the distance functions $d_X(\cdot , \cdot )$ $d_Y(\cdot , \cdot )$ are well defined due to injectivity of *f* and *g* assumed in Sect. 2.1.

7 All experiments were run on Intel Core i3 CPU with 8GB memory.

8 We remark that the barycenter problem can be defined in terms of $GW_\tau$ as a minimization of a weighted sum of $GW_\tau$ distances. However, the resulting optimal vector of geodesic distances that represents the barycenter trajectory cannot be unambiguously translated into a trajectory form.

9 The code is available in the Supplementary Information and can be used for computing FGW barycenters for trajectories of interest.
